# The species and abundance of gut bacteria both positively impact *Phortica okadai* behavior

**DOI:** 10.1186/s13071-024-06297-3

**Published:** 2024-05-11

**Authors:** Di Li, Lingjun Wang, Liang Wang, Yanting Gou, Bo Luo, Rong Yan, Hui Liu

**Affiliations:** 1 Department of Parasitology, Zunyi Medical University, Zunyi, 563000 China; 2 NHC Key Laboratory of Parasite and Vector Biology, National Institute of Parasitic Diseases, Chinese Center for Disease Control and Prevention, Shanghai, 200025 China

**Keywords:** *Phortica okadai*, Gut bacteria, Abundance, Host behavior, *Thelazia callipaeda*

## Abstract

**Background:**

Gut bacteria, which serve as essential modulators, exert a significant impact on insect physiology and behavior and have substantial application potential in pest management. The dynamics of gut bacteria and their impact on *Phortica okadai* behavior remain unclear.

**Methods:**

In this study, the dynamics of gut bacteria at different developmental stages in *P. okadai* were analyzed using 16S ribosomal RNA (rRNA) gene sequencing, and the species and abundance of gut bacteria that affect host behavior were examined via behavioral experiments.

**Results:**

A total of 19 phyla, 29 classes, 74 orders, 101 species, and 169 genera were identified. The results of the behavioral experiments indicated that the species *Lactiplantibacillus argentoratensis*, *Acetobacter tropicalis*, *Leuconostoc citreum*, and *Levilactobacillus brevis* effectively influenced the feeding preference of *P. okadai*, and the single-bacterium-seeded *P. okadai* exhibited feeding preferences distinct from those of the germ-free (GF) and wild-type *P. okadai*.

**Conclusions:**

The species and relative abundance of gut bacteria together positively impact *P. okadai* behavior. *Lactiplantibacillus argentoratensis*, as the most attractive bacteria to *P. okadai*, presents opportunities for novel pest control strategies targeting this vector and agricultural pest.

**Graphical Abstract:**

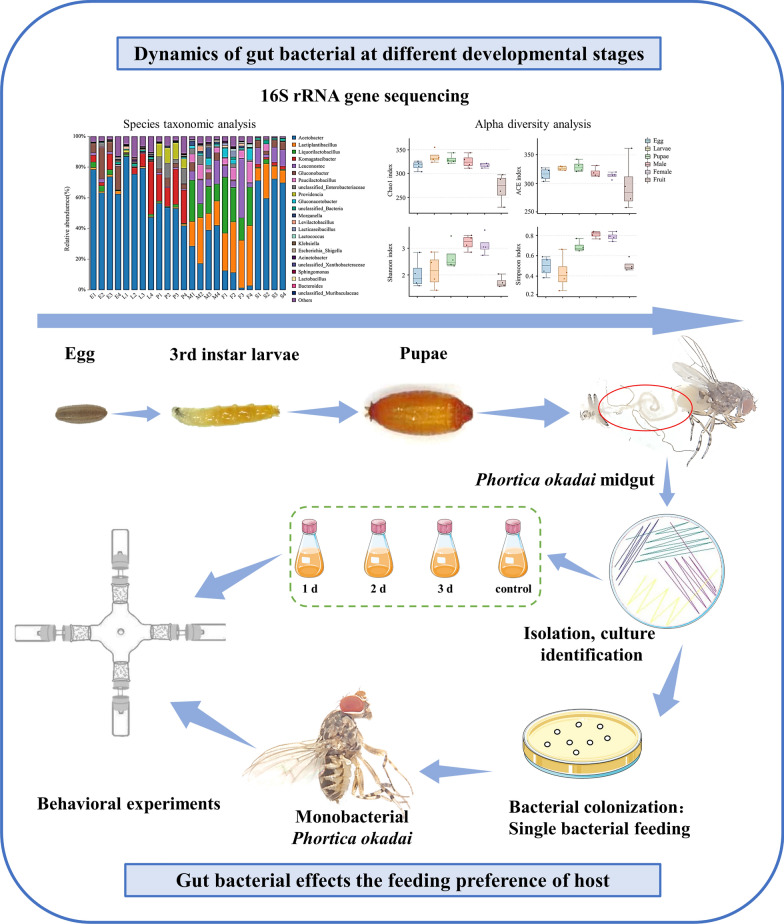

**Supplementary Information:**

The online version contains supplementary material available at 10.1186/s13071-024-06297-3.

## Background

*Phortica okadai* (Diptera: Drosophilidae), a vector of the zoonotic nematode *Thelazia callipaeda* (Spirurida: Thelazioidea) in Asia [1, 2], is a highly polyphagous pest of many commercially grown fruits, such as pear, apple, banana, citrus, and mango [3, 4]. As a significant public health concern, vector-borne *T. callipaeda* has a wide range of host species, including dogs, cats, and other mammals, as well as humans [5]. In view of the important role of *P. okadai* in agriculture and medicine, the study of its management strategies has drawn much attention in recent years. Unfortunately, to date, the control of *P. okadai* has relied mainly on insecticides, and no effective green control strategies are available.

Olfaction-based behavior manipulation technology is an environmentally friendly control method that specifically regulates the behavior of target pests and has significant application potential [6]. Attractants developed from plant volatiles and pheromones have been successfully used for many years in pest management, such as plant-derived kairomones for female *Agriotes brevis* and *Agriotes ustulatus* control, which can attract both males and females of these species by containing a blend of pheromones and plant volatiles [7]. Traps baited with SuzukiiTrap^®^ and Z-Kinol, an attractant based on acetoin and methionol, were reported for monitoring *Drosophila suzukii* populations [8]. Interestingly, convincing evidence indicates that gut bacteria play essential roles in the growth, development, and environmental adaptation of host insects [9]. In particular, in recent years, there have been numerous studies on the ability of gut bacteria to attract their hosts and influence host feeding behavior and food choice, highlighting their potential application in pest control. For example, *Enterobacter* sp. strongly attracted *Bactrocera tau* adults according to laboratory and field bioassays [10]. *Citrobacter* species were found to exhibit the greatest attraction to *Bactrocera dorsalis* [11]. The species of gut bacteria that can attract hosts have been well documented in these insects. Moreover, some research has also been conducted on whether the chemical components in the supernatant of gut bacteria can affect the host’s olfactory system and subsequently influence its behavior. As reported in Chaudhury’s research, dimethyl disulfide, a volatile component of the supernatant of *Klebsiella oxytoca*, can attract *Cochliomyia hominivorax* to feed [12].

However, whether the abundance of gut bacteria also contributes to influencing host behavior is often overlooked. The core microbiota, which generally has relatively high abundance, has been shown to play important roles in host physiology in some insects [13, 14]. Considering the aforementioned issues and the significance of abundance in ecological research, we hypothesize that the impact of gut bacteria on host behavior is not only linked to the species but also extends to the abundance of these bacteria within the host’s gut. To test this hypothesis, in our study, 16S sequencing was applied to analyze the diversity and variations in gut bacteria at various developmental stages of *P. okadai*. Subsequently, we isolated and cultured the gut bacteria from adult *P. okadai* and identified them using polymerase chain reaction (PCR). Furthermore, germ-free (GF) *P. okadai* was established and colonized with a single strain of a gut bacterium to artificially increase its abundance. Finally, behavioral experiments were conducted to assess the differential attractiveness of gut bacterial cultures to GF, single-bacterium-seeded, and wild-type *P. okadai*. This research not only contributes novel insights into the factors influencing how gut bacteria affect host behavior in insects but also offers valuable information for the development of innovative pest control strategies targeting this agricultural and medical pest.

## Methods

### *Phortica okadai* rearing and microbiota manipulation

The species used in the study was captured in a pear orchard in Zunyi, a city in Southwest China [15], and then cultured and reared at 28 ± 1 °C and 70 ± 5% relative humidity in a 14 h:8 h light/dark cycle in an artificial climate box (BIC-400, Shanghai, China). Pears that were naturally overripe (28 ± 1 °C) after 3 days were used as culture media.

The generation of GF *P. okadai* was achieved by feeding newly emerged adult individuals autoclaved pears containing a mixture of three antibiotics, ampicillin (300 µg/ml), streptomycin (300 µg/ml), and tetracycline (90 µg/ml), for 72 h in a biosafety cabinet [16]. Single-bacterium-seeded strains were produced by cultivating GF *P. okadai* with autoclaved pears inoculated with a single bacterial isolate from the fly gut using 100 µl of bacterial suspension at a density of 1.5 × 10^8^ cells/ml for 24 h in a biosafety cabinet. Each whole gut of GF or single-bacterium-seeded *P. okadai* was dissected and authenticated using Luria–Bertani (LB) medium.

### DNA extraction and amplification

Eggs, larvae, pupae, and adults of both sexes were collected and washed with 75% ethanol for 1 min, 1% sodium hypochlorite for 3 min, and sterile water three times to remove surface contaminants. Thirty adults of each sex were dissected under a stereomicroscope (Leica S9i, Germany) to isolate the midgut, frozen in liquid nitrogen, and stored at −80 °C [17].

Genomic DNA was extracted from different stages of *P. okadai* and naturally overripe pear (28 ± 1 °C) after 3 days using a TGuide S96 bead-based fecal genomic DNA extraction kit (Tiangen, DP812, China). The DNA concentration was measured using a multimode reader (Synergy HTX, BioTek Instruments, China). The V3–V4 hypervariable region of the 16S ribosomal RNA (rRNA) gene (primers 338F: 5′-ACTCCTACGGGAGGCAGCA-3′ and 806R: 5′-GGACTACHVGGGTWTCTAAT-3′) was amplified by PCR [18]. PCR was performed in a 10 µl reaction mixture containing 10 ng of genomic DNA, 0.2 µl of KOD FX Neo (Toyobo, Japan), 5 µl of KOD FX Neo buffer, 2 µl of deoxyribonucleoside triphosphate (dNTP) (2 mM), 0.3 µl of primers 338F (10 mM) and 806R (10 mM), and adequate distilled water (dH_2_O) to maintain a final volume of up to 10 µl under the following conditions: initial denaturation step of 95 °C for 5 min, followed by 25 cycles of denaturation at 95 °C for 30 s, annealing at 50 °C for 30 min, and elongation at 72 °C for 40 s, followed by 7 min final elongation at 72 °C. The DNA integrity was checked by 1.8% (w/v) agarose gel electrophoresis.

### Library construction and sequencing

The target region PCR products (10 µl) were purified by adding VAHTS™ DNA Clean Beads at a 1:1 ratio, and then barcode indexing and Illumina adapters were added by Solexa PCR (20 µl reaction volumes), which used 5 µl of pooled PCR product, 2.5 µl of each index (forward and reverse), and 10 µl of 2× Q5 High-Fidelity Master Mix (NEB, USA) [19]. The PCR conditions were 98 °C for 30 s, followed by 10 cycles of 98 °C for 10 s, 65 °C for 30 s, and 72 °C for 30 s, and a final extension step of 5 min at 72 °C. Agarose gel electrophoresis was performed on a 1.8% (w/v) agarose gel, and the results were quantified by the ImageJ program. Each sample was mixed by aspirating 150 ng, and the mixed samples were purified before gel cutting using the Cycle Pure Kit and recovered by the Monarch DNA kit. The final gene library was evaluated on a Qsep400 (BiOptic, Taipei, Taiwan) for concentration and quality and then sequenced on an Illumina NovaSeq 6000. The 16S rRNA gene library construction and sequencing were completed by Biomarker Technologies Corporation (Beijing, China).

### Processing of sequence data and bioinformatics analysis

Using Trimmomatic v0.33 software, the raw reads obtained from sequencing were filtered. The paired-end fastq files were removed using VSEARCH v2.8.1 [20]. Then, we used UCHIME v4.2 software to identify and remove the chimeric sequences to obtain the final effective reads. Sequences were removed from inclusion according to the following criteria: the average mass of bases was less than 20, the sequences were less than 350 base pairs (bp) in length, the sequences contained primer mismatches, the reads were low quality, and the sequences could not be spliced. Clustering of the reads was performed at a 97.0% similarity level to obtain operational taxonomic units (OTUs), and the OTUs were taxonomically annotated based on the Silva taxonomic databases.

Venn diagrams were used to show the number of common, unique features between samples for groups with bacterial abundance percentages higher than 0.1% (http://www.ehbio.com/test/venn/#/). The community composition of each sample was determined at each level (phylum, genus) and mapped using R tools (http://www.cloud.biomicroclass.com/CloudPlatform/home). The alpha diversity was calculated using species-level annotation information statistics, and the Wilcoxon test was used to analyze the variability between groups, with *P* < 0.05 indicating a significant difference. The number of sequences versus the number of species was used to construct a rarefaction curve. UPGMA (unweighted pair-group method with arithmetic means) analysis was based on the Bray‒Curtis distance between samples. The results of principal coordinate analysis (PCoA) and non-metric multidimensional scaling (NMDS) analysis were plotted separately using the online website https://www.biocloud.net/, and permutational multivariate analysis of variance (PERMANOVA) was used to test whether beta diversity was significantly different between samples of different groups, with *P* < 0.05 indicating a significant difference. Analysis of variance (ANOVA), a nonparametric statistical method (*P* < 0.05), was used to test the mean values of multiple samples at the genus level to determine the significance of the differences in the mean values of samples at different developmental stages.

### Isolation, identification, and culture of bacteria from *P. okadai* guts

Under a postural dissector (S9i, Leica, Wetzlar, Germany), three males and three females of *P. okadai* were fed normally, and the intestine was dissected using the abovementioned method. Phosphate-buffered saline (PBS) solution (0.5 ml) was added to the centrifuge tube, and the mixture was ground. Then, 10, 10^2^, 10^3^, 10^4^, and 10^5^ dilutions were obtained, and 50 µl of the diluted solution was coated on a blood plate (90 mm) and cultured for 24 h, 48 h, and 72 h (37 °C, 5% CO_2_ atmosphere). According to the growth characteristics of the colonies on the plates, different forms of bacteria were isolated, cultured, and purified twice via a zoning line.

Bacterial genome extraction was performed using a Bacterial Genomic DNA Kit (ZP301, Zoman Biotechnology, Beijing, China). PCR was performed using the general 16S primers 27F (5′-AGAGTTTGATCCTGGCTCAG-3′) and 1492R (5′-GGTTACCTTGTTACGACTT-3′) [21], followed by sequencing (Tsingke, Chongqing, China). The PCRs contained 12.5 µl of K5 HiFi 2× PCR Master Mix (ZT211), 1 µl of template DNA, and 1 µl of primers in a total volume of 25 µl. The cycling conditions were 95 °C for 5 min; 30 cycles of 94 °C for 30 s, 56 °C for 30 s, and 72 °C for 60 s; and a final extension at 72 °C for 5 min. The PCR products were sequenced by an ABI 3730xl DNA Analyzer (Fuzhou, China) with both strands using the 27F-1492R primer. The sequences of different bacterial isolates were analyzed using EZBioCloud (https://www.ezbiocloud.net/) [22] to select the most homologous 16S ribosomal DNA (rDNA) sequences.

### Behavioral experimental design

Four species of gut bacteria, *Lactiplantibacillus argentoratensis*, *Lysinibacillus fusiformis*, *Leuconostoc citreum*, and *Levilactobacillus brevis*, were cultivated on de Man–Rogosa–Sharpe (MRS) medium (Solarbio, Beijing, China) in 12 ml culture tubes (Natural, Dual Cap, Sterile) under the following conditions: 20 µl of bacterial suspension at a density of 1.5 × 10^8^ colony-forming units (CFU)/ml was added to 2.5 ml of medium and incubated for 24 h, 48 h, and 72 h at 37 °C and 180 r/min. The same method was used to culture another bacterium, *Acetobacter tropicalis*, but with a custom medium consisting of 10 g of glucose, 10 g of yeast powder, and 1000 ml of deionized water, which was autoclaved for 20 min, and then 40 ml of anhydrous ethanol was added.

To evaluate the species of gut bacteria that can attract hosts, media containing individual bacterial species cultured for varying durations were subjected to behavioral experiments with a four-armed attraction device on wild-type *P. okadai*. The most attractive medium was then selected for further verification with GF *P. okadai* to differentiate between the wild-type and GF strains. Moreover, to explore whether the abundance of gut bacteria also plays a role in influencing host behavior, single-bacterium-seeded *P. okadai* were subjected to behavioral experiments using the most attractive medium by the same method.

Additionally, to test the attractiveness of the bacterial fermentation liquid and autoclaved supernatants, the most attractive bacterial cultures were subsequently transferred to a 2 ml tube and labeled as bacterial fermentation liquid, and then, the residual bacterial cultures were centrifuged at 10,000 rpm for 20 min. Afterward, 2 ml of the resulting supernatants were autoclaved at 121 °C for 20 min and denoted as autoclaved supernatants. The MRS medium and live bacteria were used as controls. Ultimately, to evaluate the potential application of the most attractive bacterial cultures, 3-day naturally overripe pears were used as a reference.

All behavioral experiments were conducted using a four-armed olfactometer or a Y-tube bioassay. *Phortica okadai* individuals were starved (only water was provided) for 12 h before the beginning of the experiments, and the results were recorded after an additional 12 h. Each experiment utilized 30 *P. okadai* specimens with 10 replicates to ensure statistical robustness.

### Statistical analysis

The data were tested for a normal distribution and subsequently analyzed for multiple (one-way ANOVA, least significant difference [LSD] post hoc test) or two-group (Student’s *t*-test, *P* < 0.05) treatments using GraphPad Prism 9. These differences were considered significant at the *P* < 0.05 level.

## Results

### Sequencing data of 16S rRNA

A total of 1,919,824 read pairs were obtained by sequencing 24 samples from six groups. A total of 1,913,172 clean reads were generated after double-ended read quality control and splicing, generating at least 79,205 clean reads per sample and an average of 79,715 clean reads (details of each sample are provided in Additional file 1: Table S1). After clustering at the 97% similarity level, 313 OTUs, 339 OTUs, 341 OTUs, 344 OTUs, 342 OTUs, and 339 OTUs were obtained for each sample (Additional file 1: Figure S1), generating a total of 346 OTUs. The rarefaction curve showed a gradual decrease in the latter part of the curve, which suggested that the number of clones sampled was sufficient to provide an adequate estimation of all the samples (Additional file 1: Figure S2).

### Dynamics of gut bacteria at different developmental stages

**Fig. 1:**
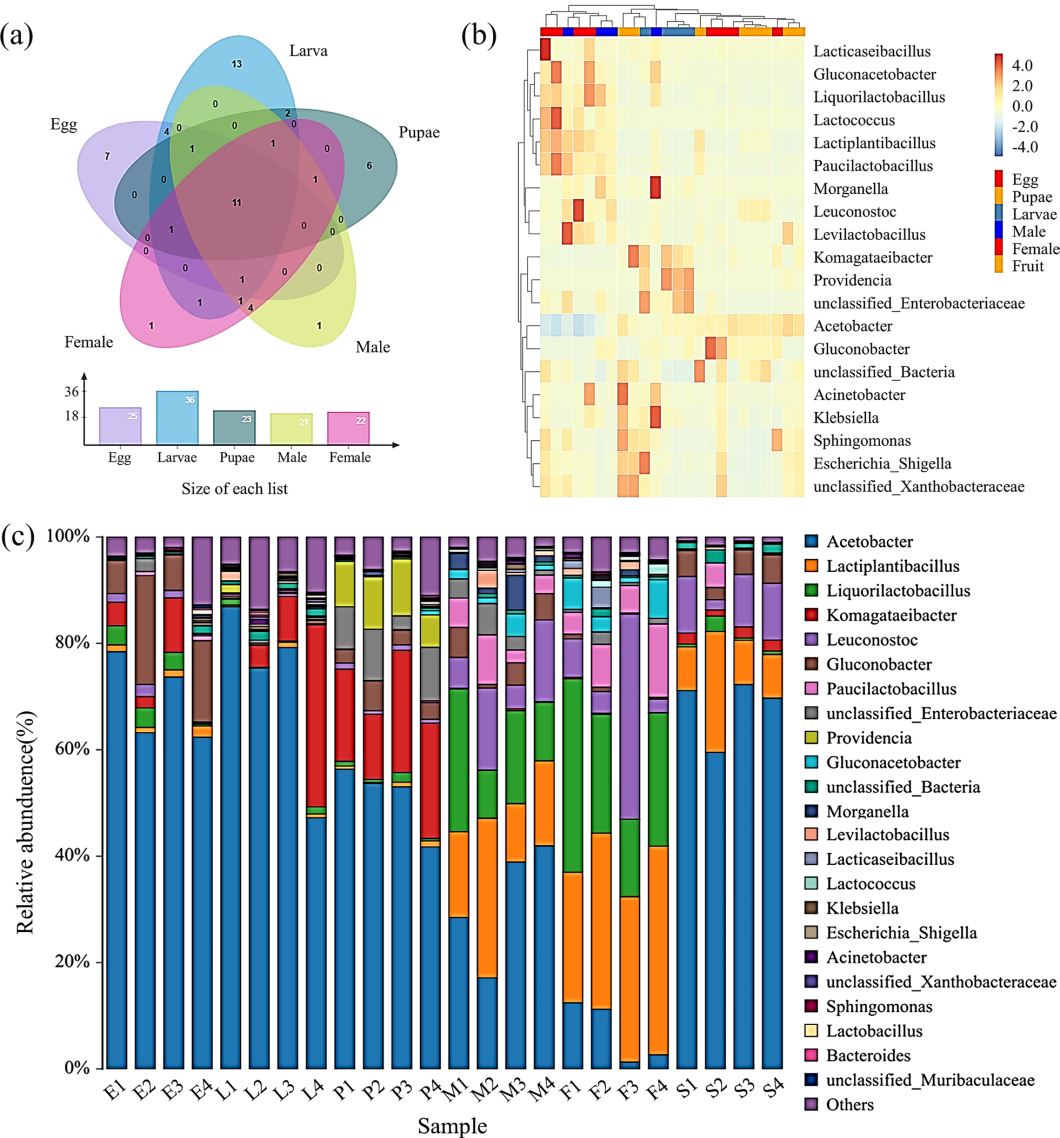
Taxonomic analysis of different developmental stages of *P. okadai* and overripe fruits at the genus level. **a** Venn diagrams of common bacteria of *P. okadai*. **b** A heatmap of species abundance was constructed by clustering the top 20 species. **c** Microbiome composition (E: egg, L: larvae, P: pupae, M: male midgut, F: female midgut, and S: overripe pear)

The percentage of gut bacterial abundance in *P. okadai* at different developmental stages varied slightly, with 54 genera detected in the samples of species constituting more than 99.9% of the abundance of *P. okadai* (Fig. 1a), with 11 genera contained in different developmental stages, as the core bacteria, including five each in the phyla Firmicutes and Proteobacteria, and one unclassified bacterium (OTU taxonomic comparison in Additional file 1: Table S2). The bacterial composition of the adult *P. okadai* gut differed from that of the egg, larval, and pupal stages, and there were also differences in the composition of the gut bacteria between females and males. The bacteria of *P. okadai* eggs, larvae, pupae, and overripe fruits consisted mainly of Proteobacteria and, to a lesser extent, Firmicutes, in addition to a small number of Bacteroidota, Acidobacteriota, and Actinobacteriota (Additional file 1: Figure S3). In the *P. okadai* gut, Firmicutes and Proteobacteria are predominant.

At the genus level, heatmap analysis (Fig. 1b) revealed that three stages, namely, the egg, larva, and pupa stages, contained more *Acetobacter*. The abundance of *Gluconobacter* in eggs, *Enterobacteriaceae* in larvae, and *Providencia* and *Komagataeibacter* in pupae was greater than their abundance in other stages (the relative abundance of bacteria at the genus level in each sample is shown in Additional file 1: Table S3, Figure S1). Clustering analysis of the top 20 species in terms of abundance at the genus level via a species abundance heatmap (Fig. 1c) revealed that the three stages of eggs, larvae, and pupae contained large amounts of *Acetobacter* and *Lactiplantibacillus* (*P* < 0.05, Additional file 1: Figure S4). Species abundance in the gut bacteria of adult *P. okadai* is highly variable relative to that in other stages. The abundance of *Acetobacter*, *Lactiplantibacillus*, *Liquorilactobacillus*, and *Leuconostoc* was greater in the gut of adult *P. okadai*, and there were differences in the abundance of *Acetobacter* and *Lactiplantibacillus* in the gut microbiota of male and female *P. okadai*. Overripe fruits had a high abundance of *Acetobacter*, *Lactiplantibacillus*, and *Leuconostoc*. Interestingly, the highest abundance of *Acetobacter* was found in overripe fruits, while the lowest abundance of *Acetobacter* was found in the gut of adult *P. okadai* fed overripe fruits compared to other stages.

### Alpha and beta diversity analysis

**Fig. 2:**
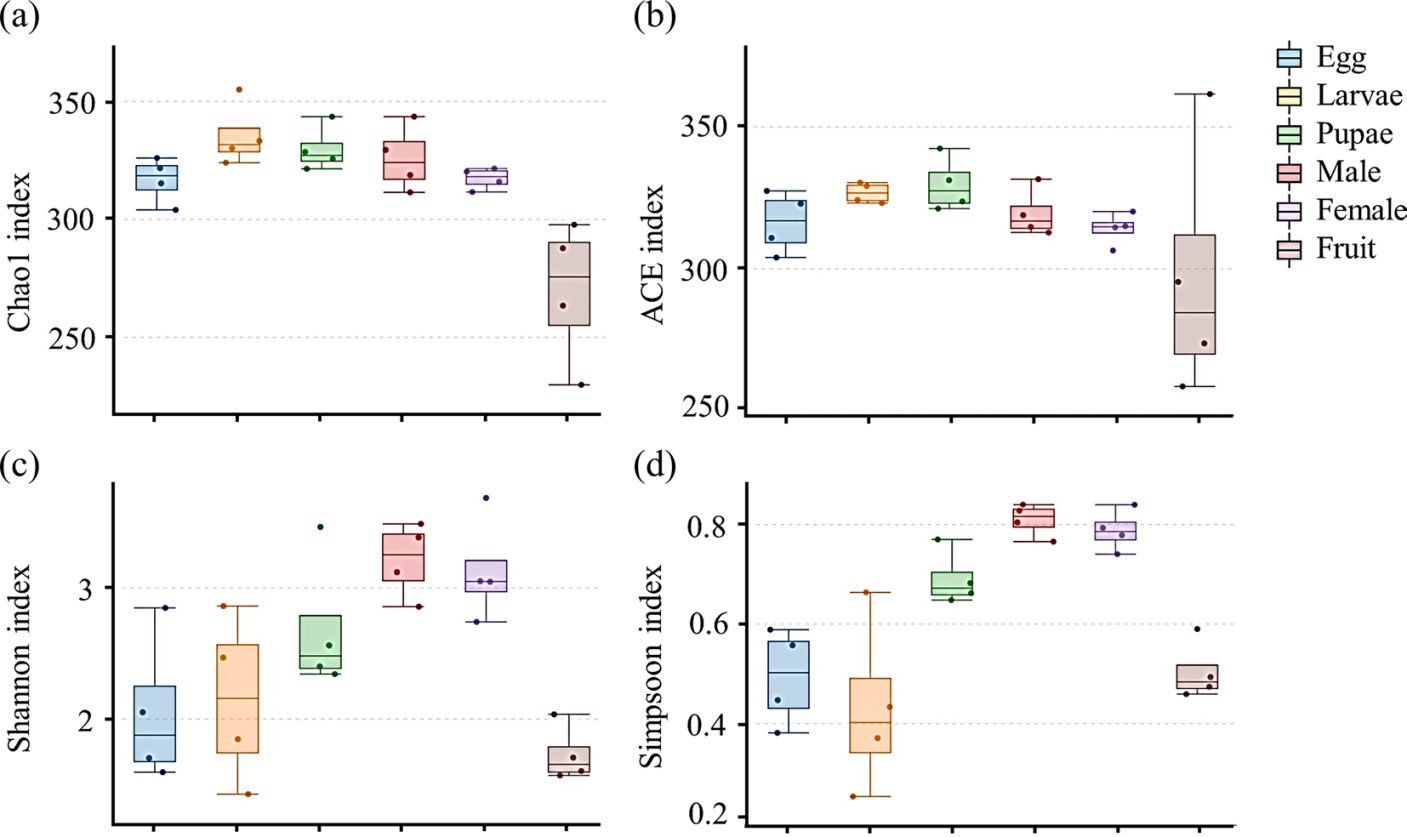
Alpha diversity of *P. okadai* fruits at different developmental stages. **a** Chao1 index, **b** ACE index, **c** Shannon index, **d** Simpson index

The Chao1 (Fig. 2a) and abundance-based coverage estimator (ACE) (Fig. 2b) indices showed relatively high richness, which indicated that *P. okadai* had significantly greater species richness than did the overripe fruits at all stages (*P* < 0.05). Both the Shannon (Fig. 2c) and Simpson (Fig. 2d) diversity indices (evenness) showed greater diversity in the developmental stages of *P. okadai* than in those of overripe fruits (*P* < 0.05), and these indices showed a gradually increasing trend. In addition, alpha index analysis revealed that overripe fruits had lower bacterial abundance and diversity than *P. okadai* at other developmental stages (alpha diversity index values for each sample are shown in Additional file 1: Table S4).

**Fig. 3:**
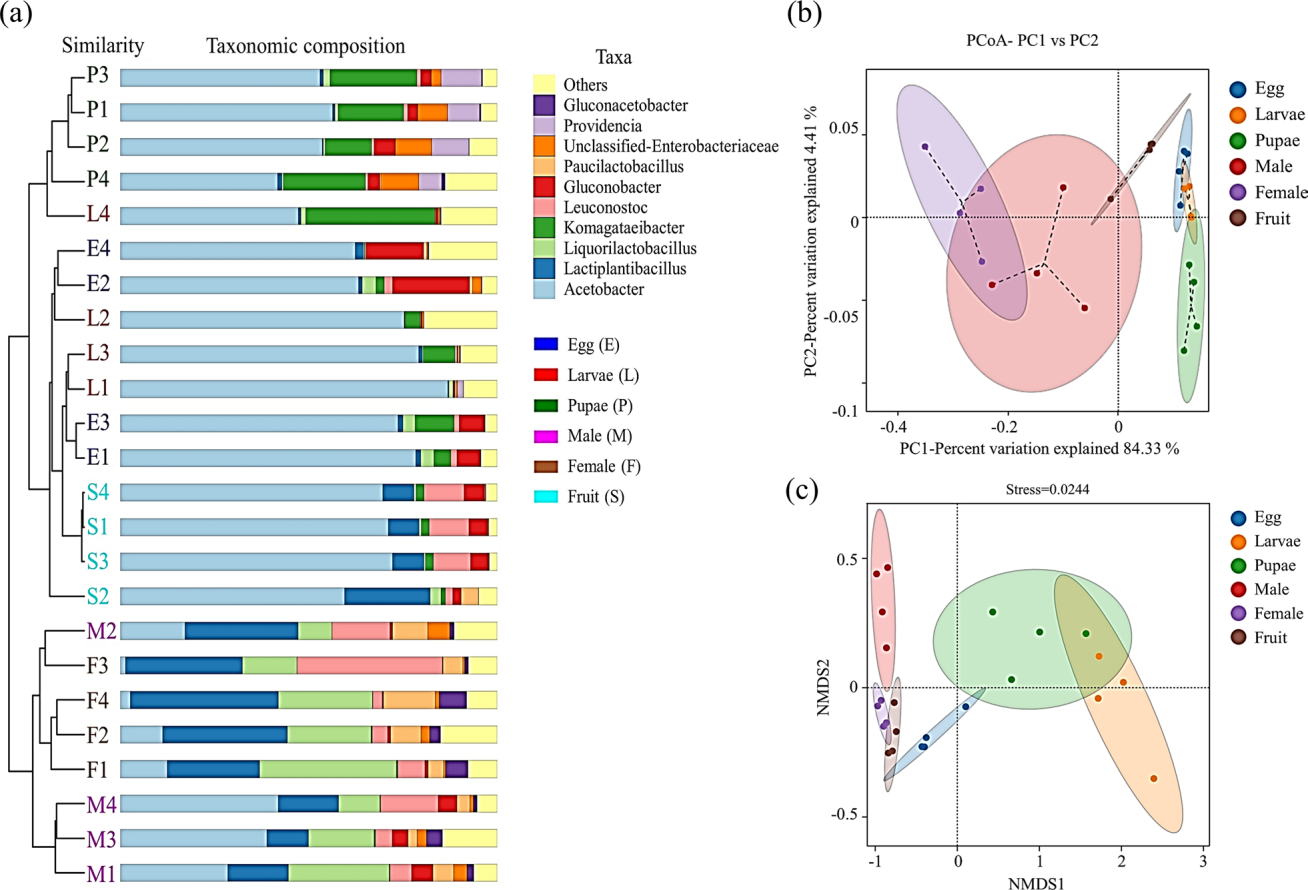
Beta diversity analysis between different developmental stages of *P. okadai* and overripe fruits at the genus level. **a** UPGMA analysis based on the Bray‒Curtis distance, **b** PCoA analysis based on the phylogenetic weighted UniFrac principle, and PERMANOVA test results. The percentage of variation explained by the principal coordinate PC1 was 84.33%, **c** NMDS analysis based on the phylogenetic weighted UniFrac principle, and PERMANOVA test results, stress = 0.0244

Beta diversity analysis revealed that the six groups of samples were divided into two branches, in which the female and male *P. okadai* samples were grouped into one branch, and the other branch comprised other developmental stages and overripe fruits (Fig. 3a). The PCoA (Fig. 3b) and NMDS (Fig. 3c) analysis results showed good aggregation of samples in different groups with little overlap of confidence intervals after clustering across developmental stages. This indicates that the bacterial colony composition at different developmental stages is similar, but there are also differences in bacterial composition among developmental stages. Statistical analysis of the beta diversity using PERMANOVA revealed statistically significant differences (*P* < 0.05).

### Bacterial isolation, identification, and culture

Based on 16S rRNA gene sequencing analysis, 14 different bacterial isolates spanning two phyla, eight families, and 12 genera were identified from adult *P. okadai* (Additional file 1: Table S5). Following the sequence alignment of the isolated bacteria, the similarity among bacterial sequences exceeded 98%, with completeness surpassing 90%, which is indicative of robust alignment outcomes. All isolated bacteria fell within the Firmicutes and Proteobacteria phyla, representing the dominant taxa observed in the 16S (V3–V4) sequencing results (Additional file 1: Figure S3).

### Laboratory attractiveness bioassays

**Fig. 4:**
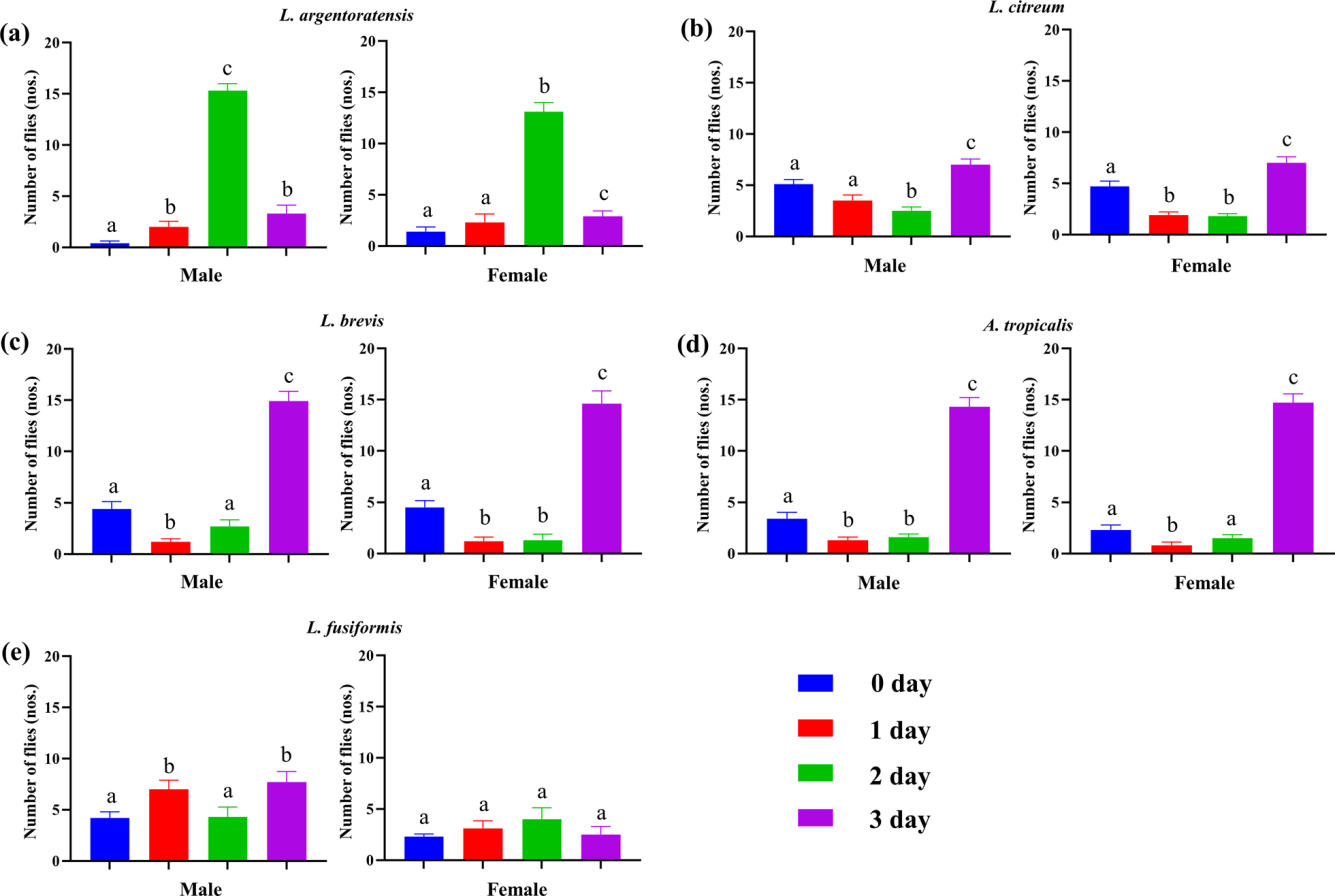
Attraction experiments on *P. okadai* on different days of bacterial culture. **a** *L. argentoratensis*. **b** *L. citreum*. **c** *L. brevis*. **d** *A. tropicalis*. **e** *L. fusiformis*. Attraction experiments for optimal culture days for four types of bacteria with attractive effects. Values marked with the same letter indicate nonsignificant differences, while those marked with different letters indicate significant differences (mean ± standard error of the mean [SEM], *n* = 10, *P* < 0.05)

Based on the results of 16S rRNA sequencing, we selected *L. fusiformis*, *L. citreum*, *L. brevis*, *L. argentoratensis*, and *A. tropicalis* as candidates for the* P. okadai* attraction experiment (Fig. 4). The experimental results showed that the five bacterial culture products had a certain attraction effect. Compared with the culture medium on day 0, both male and female fruit flies were more attracted to the culture medium supplemented with *L. argentoratensis* (Fig. 4a) on day 2 and to the culture medium supplemented with *L. citreum* (Fig. 4b)*, L. brevis* (Fig. 4c) and *A. tropicalis* (Fig. 4d) on day 3. Male *P. okadai*, but not female *P. okadai*, were attracted to the culture medium supplemented with *L. fusiformis* (Fig. 4e) on day 3.

**Fig. 5:**
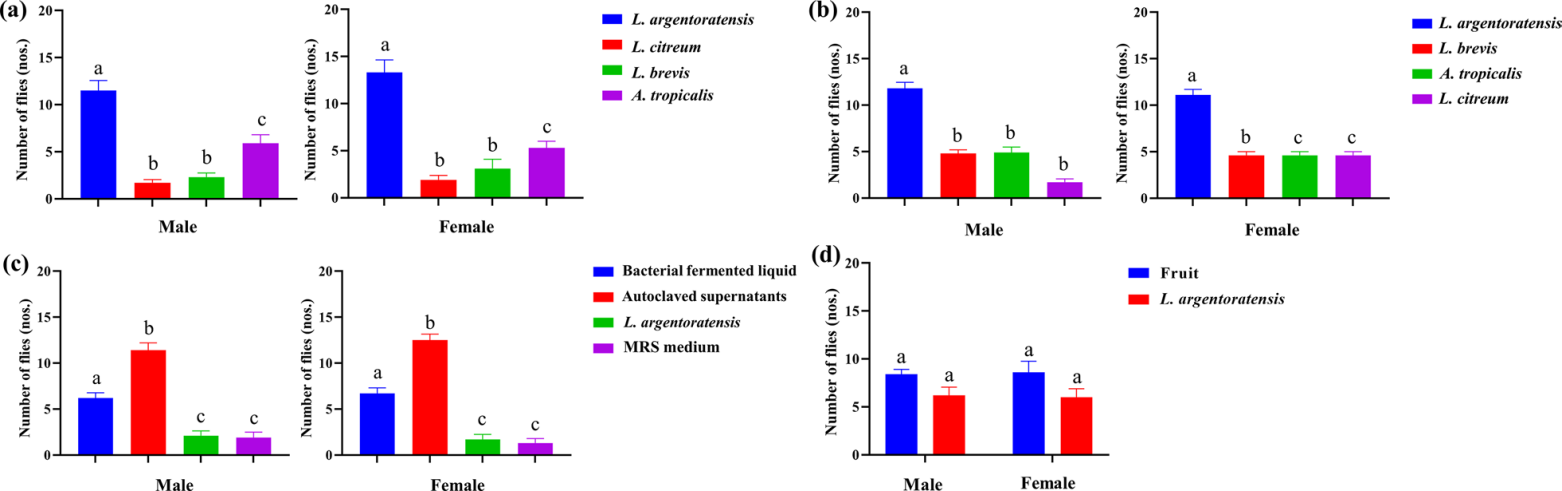
Attraction experiments on *P. okadai*. **a** Attraction experiments for optimal culture days for four types of bacteria with attractive effects. **b** Behavioral experiment of four bacterial cultures on GF specimens. **c** Different components of the bacterial culture media used in the behavioral temptation experiment. Autoclaved supernatants were cultured for 2 days, after which the *L. argentoratensis* medium was centrifuged under high pressure. Bacterial fermentation liquid: *L. argentoratensis* cultured for 2 days in culture medium was directly sterilized under high pressure. *L. argentoratensis*: bacteria that were cultured for 2 days in *L. argentoratensis* culture media and were subsequently washed after centrifugation. MRS medium: control group. **d** Overripe fruits and *L. argentoratensis* induced by the Y-tube experiment. Values marked with the same letter indicate nonsignificant differences, while those marked with different letters indicate significant differences (mean ± standard error of the mean [SEM], *n* = 10, *P* < 0.05)

Two-day cultures of *L. argentoratensis*, *L. citreum*, and *L. brevis* and 3-day cultures of *A. tropicalis* were utilized for the attraction experiment. The results demonstrated that *L. argentoratensis* exhibited the greatest attraction (Fig. 5a). An examination of the feeding preferences of GF specimens on various bacterial cultures revealed a continued preference for feeding containing *L. argentoratensis* (Fig. 5b). Subsequent behavioral attraction experiments on *P. okadai* involved different components of *L. argentoratensis* culture products to elucidate the factors attracting *P. okadai*. The findings indicated that bacterial culture under high pressure had superior effects on attracting *P. okadai* to the supernatant after bacterial culture, with both demonstrating heightened attraction effects (Fig. 5c). Notably, there was no significant difference in the preference between the overripe pear and *L. argentoratensis* for attracting *P. okadai* (Fig. 5d) (*P* < 0.05).

**Fig. 6:**
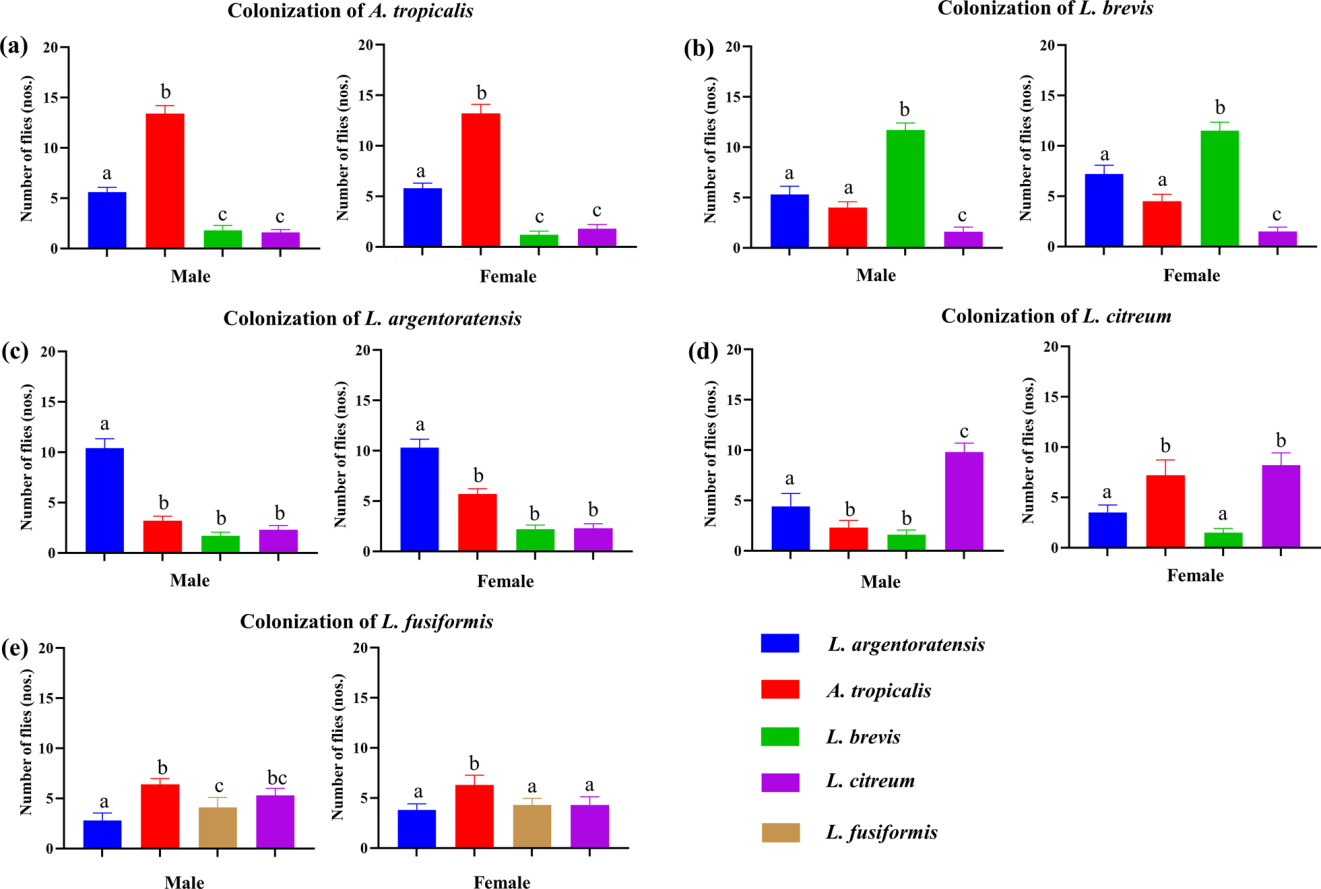
Attraction experiments on *P. okadai* with single bacterial colonization. **a** GF specimens treated with *A. tropicalis*. **b** GF specimens treated with *L. brevis*. **c** GF specimens treated with *L. argentoratensis*. **d** GF specimens treated with *L. citreum*. **e** GF specimens treated with *L. fusiformis*. Values marked with the same letter indicate nonsignificant differences, while those marked with different letters indicate significant differences (mean ± standard error of the mean [SEM], *n* = 10, *P* < 0.05)

To explore the potential influence of gut bacterial abundance on host behavior, *A. tropicalis*, *L. brevis*, *L. argentoratensis*, *L. citreum*, and *L. fusiformis* were used to colonize GF *P. okadai* for attraction experiments (Fig. 6). The experimental findings indicate that when a single bacterium colonizes the gut of *P. okadai*, the host demonstrates a preference for consuming food containing that specific bacterium.

## Discussion

Numerous studies have demonstrated that gut bacteria have various impacts on insect physiology [23]. However, although many microbial communities in the alimentary canals of insects have been described, there is currently limited research on vectors, especially Steganinae, in which some species act as vectors of *T. callipaeda*. Therefore, in this study, we first surveyed the microbial composition of *P. okadai* to determine how the gut microbiota may play a role in growth and development via 16S rRNA gene sequencing. Subsequently, the gut bacteria from adult *P. okadai* were cultured and used to conduct a behavioral experiment with GF, single-bacterium-seeded, and wild-type *P. okadai*, which were designed to evaluate the influence of both the species and abundance of gut bacteria on host behavior. Simultaneously, we conducted an initial exploration of the attractant components in gut bacterial cultures influencing host behavior.

Eleven core bacteria present in all developmental stages of *P. okadai* were identified and described in recent studies as playing an important role in insect physiology (Fig. 1a and Additional file 1: Table S2). *Acetobacter*, which had the greatest abundance in eggs, larvae, pupae, and males (Fig. 1b, c and Additional file 1: Table S3), contains multiple isoleucine, valine, and leucine biosynthetic genes, and these are essential amino acids for insects [24, 25]. *Liquorilactobacillus* and *Lactiplantibacillus*, which are more abundant in adults (Fig. 1b, c and Additional file 1: Table S3), are commonly considered to be human, symbiotic, and beneficial bacteria in animals and are involved in trophic metabolism and antimicrobial action [26, 27]. *Lactiplantibacillus* bacteria have also been shown to produce bacteriocins, which have good antibacterial activity against food-borne spoilage bacteria and pathogens in food, maintaining the relative stability of the composition structure of the bacteria in the insect gut [28]. *Providencia*, which has the greatest abundance in pupae (Fig. 1b and Additional file 1: Table S3), produces regulatory neurotransmitters that have been proposed to be used to regulate nervous system activity and behavior in *C. elegans* [29, 30]. Furthermore, studies on *Bactrocera minax* have shown that *Providencia* species have diapause effects and can help insects survive harsh winters [31–33]. In addition, *Klebsiella* and *Enterobacteriaceae* bacteria were found in *Heliconius erato* and *Bussiola fusca* to produce enzymes for the breakdown of lignin, cellulose and plant-derived compounds, thus facilitating the digestion of food by the host (Fig. 1b and Additional file 1: Table S3) [34, 35].

Previous studies have shown that feeding characteristics are the primary factor affecting the structure of the gut bacterial communities of insects [36, 37]. The horizontal transmission of food has a significant effect on the bacterial structure of the insect gut, and some bacteria are not able to colonize the body in the absence of external bacterial supplementation, causing changes in the dominant species or core bacterial groups at different life stages [38]. In this study, the abundance of *Acetobacter*, which is highly abundant in overripe fruits, decreased in adult *P. okadai*, while, in contrast, the abundance of *Liquorilactobacillus* increased significantly (Fig. 1b and Additional file 1: Table S3). Moreover, *P. okadai* is a multi-food species, and adults are exposed to a relatively great diversity of bacterial species in the surrounding environment, which explains the greater alpha diversity index in the adult stage than in the other stages (Fig. 2c, d). On the other hand, vertical transmission of bacteria also plays an important role in intestinal bacterial proliferation [39]. The structure of the gut bacteria in holometabolous insects, which undergo complete restructuring of the body during metamorphosis, changes because of the reorganization of the foregut and hindgut. The dynamics of gut bacteria at different developmental stages may be a result of the long-term evolution of insects adapting to various food sources and ecological niches [40, 41]. Eleven core bacteria identified in *P. okadai* (Fig. 1a and Additional file 1: Table S3) could be the most likely candidates for vertical transmission, but further research based on core genome phylogenetic analysis is needed.

Gut bacterial species with the ability to attract hosts have been identified in many insects [42], which was corroborated in our study. The behavioral experiment results indicated that the bacterial culture products of different bacteria and different cultivation durations had varying effects on the feeding behavior of *P. okadai* (Fig. 4). Both normally reared and GF *P. okadai* showed a preference for food containing *L. argentoratensis* (Fig. 5a, b). Interestingly, a novel finding that the abundance of gut bacteria also mediates a positive impact on host behavior was validated through behavioral experiments with GF and single-bacterium-seeded *P. okadai*. All the single-bacterium-seeded *P. okadai* specimens exhibited feeding preferences distinct from those of the GF and wild-type specimens (Fig. 6). Although the colonization of *P. okadai* by *L. fusiformis* did not manifest as pronounced behavioral changes compared to that of the other four bacterial strains, to a certain extent, the influence of bacterial abundance on host behavior is still unclear. This is evidenced by the fact that colonization with *L. fusiformis* did not exhibit the same heightened preference for *L. argentoratensis* culture observed in GF and wild-type *P. okadai* (Fig. 6).

Despite numerous studies, a definitive theoretical framework explaining the mechanisms through which gut bacteria influence host behavior remains elusive, which is one area on which future research efforts should focus. At present, substantial evidence suggests that the attraction of hosts to gut bacterial cultures is primarily linked to the metabolites produced by the gut bacteria [43–45]. For example, volatiles extracted from trypticase soy broth cultured with *Staphylococcus aureus* can attract adult Mexican flies [46]. Behavioral assays revealed that both adults and larvae of *D. melanogaster* were attracted to the headspace of *Saccharomyces cerevisiae* and *Lactobacillus plantarum* [47]. In our study, the bacterial fermentation liquid exhibited greater attraction to *P. okadai* than did the autoclaved supernatants, which may be attributed to the release of intracellular metabolites when the bacterial culture was subjected to high-pressure treatment (Fig. 5c). Concurrently, there is evidence suggesting that the olfactory system of insects contributes to the modulation of host behavior by gut bacteria. Similarly, in the Colorado potato beetle, a reduction in gut microbes could influence the expression levels of LdecOR9 and LdecOR17 [46]. As a critical element within the olfactory system, odorant receptors (ORs) play a pivotal role in the recognition of volatile compounds, exerting a key influence on the modulation of insect behavior [48]. Although various perspectives have been proposed to elucidate the mechanisms through which the gut microbiota regulates the expression of host olfactory receptors, the precise mechanisms involved remain inadequately elucidated. For example, the metabolic byproducts resulting from microbial metabolic exchange have been identified as key compounds determining olfactory preferences in *Drosophila* [49]. Metabolites from gut bacteria are transformed by intestinal epithelial cells and transmitted through neural pathways to specific brain regions, increasing the expression of relevant taste signals and leading to preferential feeding behavior [50, 51]. Alternatively, this may be due to related metabolites passing through the intestine into the lymphatic circulation and reaching olfactory receptors, enhancing the recognition of bacterial volatiles and related gene expression and thus affecting the feeding preference of the host [48, 49, 52].

However, despite extensive experimentation and analysis conducted in the present study, there were several limitations, as the present investigation suffers from an absence of extensive interspecies corroboration, extending to various taxonomic groups, encompassing paradigmatic organisms such as *D. melanogaster*, among others. Moreover, to date, most conclusions predominantly rely upon laboratory evidence, but a greater number of field-based studies are warranted to authenticate and substantiate these findings, and the specific metabolites of gut bacteria, such as *L.* *argentoratensis*, that are responsible for influencing the behavior of *P. okadai* remain unidentified, necessitating further research endeavors to clarify the molecular basis of the impacts of these gut bacteria on host behavior.

## Conclusions

In conclusion, in this study, the dynamics of gut bacteria at different developmental stages of *P. okadai* were first explored via 16S rRNA gene sequencing, and 11 core bacteria were identified. Behavioral experiments have shown that the species and abundance of gut bacteria jointly positively impact *P. okadai* behavior, and *L. argentoratensis* cultures are the most attractive to *P. okadai*, which presents opportunities for novel pest control strategies targeting this vector and agricultural pest.

### Supplementary Information

**Additional file 1: Figure S1.** Clustering at the 97.0% similarity level and the number of outs for each group of overripe fruits and *P. okadai* fruits at different developmental stages. **Figure S2.** Rarefaction curves based on OTU numbers at different developmental stages of *P. okadai* and overripe fruits. **Figure S3.** Taxonomic composition and relative abundance of symbiotic bacteria at the phylum and genus levels between different developmental stages of *P. okadai* and overripe fruits. **Figure S4.** Differences in the microbial community abundance of *P. okadai* at different developmental stages and of overripe fruits at the genus level according to analysis of variance (ANOVA, Benjamini–Hochberg false discovery rate [BH-FDR]). The figure shows the bacterial genera with the highest percentages of abundance. **Table S1.** The sequencing data for the overripe fruits and *P. okadai* at different developmental stages in each sample is shown in the following table: raw reads are the number of raw reads obtained from sequencing; clean reads are the number of high-quality reads obtained after raw sequence quality control; effective reads are the number of effective sequences after cleaning reads by splicing (double-end), filter length and chimeras; and AvgLen (bp) is the average sequence length of the sample. (E: egg, L: larvae, P: pupae, M: male midgut, F: female midgut, and S: overripe pear). **Table S2.** Fifty-four genera were detected in species with a *P. okadai* abundance of more than 99.9%, of which 11 genera are present at different developmental stages and are known as the core microbiota. **Table S3.** Relative abundance (%) of taxa associated with overripe fruits and *P. okadai* at different developmental stages at the genus level. **Table S4.** Alpha diversity of the gut microbiota of *P. okadai* at different developmental stages and overripe fruits. The Chao1 diversity estimator, the number of OTUs, and the Shannon and Simpson indices were estimated for all the samples (E: eggs, L: larvae, P: pupae, M: male midgut, F: female midgut, S: overripe fruits). **Table S5.** BLAST-based alignment of 16S rRNA sequences from the intestinal tract of adult *P. okadai*.

## Data Availability

The raw 16S rRNA gene sequencing data supporting the conclusions of this study have been made available at NCBI (https://www.ncbi.nlm.nih.gov) under project number PRJNA950239, and all other study data are included in the article and/or supporting information.

## Abbreviations

rRNA: Ribosomal RNA
PCR: Polymerase chain reaction
LB: Luria–Bertani
dH_2_O: Distilled water
dNTP: Deoxyribonucleoside triphosphate
UPGMA: Unweighted pair-group method with arithmetic means
PCoA: Principal coordinate analysis
NMDS: Non-metric multidimensional scaling
PBS: Phosphate-buffered saline
rDNA: Ribosomal DNA
OTUs: Operational taxonomic units
GF: Germ-free
ORs: Odorant receptors
