# Supplementary material for: The species and abundance of gut bacteria both positively impact Phortica okadai behavior
Source: Parasit Vectors. 2024 May 11;17:217. doi: 10.1186/s13071-024-06297-3 (PMC11088764; doi:10.1186/s13071-024-06297-3)
Supplement: Supplementary file 1 — Additional file 1: Figure S1. Clustering at the 97.0% similarity level and the number of outs for each group of overripe fruits and P. okadai fruits at different developmental stages. Figure S2. Rarefaction curves based on OTU numbers at different developmental stages of P. okadai and overripe fruits. Figure S3. Taxonomic composition and relative abundance of symbiotic bacteria at the phylum and genus levels between different developmental stages of P. okadai and overripe fruits. Figure S4. Differences in the microbial community abundance of P. okadai at different developmental stages and of overripe fruits at the genus level according to analysis of variance (ANOVA, Benjamini–Hochberg false discovery rate [BH-FDR]). The figure shows the bacterial genera with the highest percentages of abundance. Table S1. The sequencing data for the overripe fruits and P. okadai at different developmental stages in each sample is shown in the following table: raw reads are the number of raw reads obtained from sequencing; clean reads are the number of high-quality reads obtained after raw sequence quality control; effective reads are the number of effective sequences after cleaning reads by splicing (double-end), filter length and chimeras; and AvgLen (bp) is the average sequence length of the sample. (E: egg, L: larvae, P: pupae, M: male midgut, F: female midgut, and S: overripe pear). Table S2. Fifty-four genera were detected in species with a P. okadai abundance of more than 99.9%, of which 11 genera are present at different developmental stages and are known as the core microbiota. Table S3. Relative abundance (%) of taxa associated with overripe fruits and P. okadai at different developmental stages at the genus level. Table S4. Alpha diversity of the gut microbiota of P. okadai at different developmental stages and overripe fruits. The Chao1 diversity estimator, the number of OTUs, and the Shannon and Simpson indices were estimated for all the samples (E: eggs, L: larvae, P: [file 13071_2024_6297_MOESM1_ESM.docx]

**Fig. S1** Clustering at the 97.0% similarity level and the number of outs for each group of overripe fruits and *P. okadai* fruits at different developmental stages.


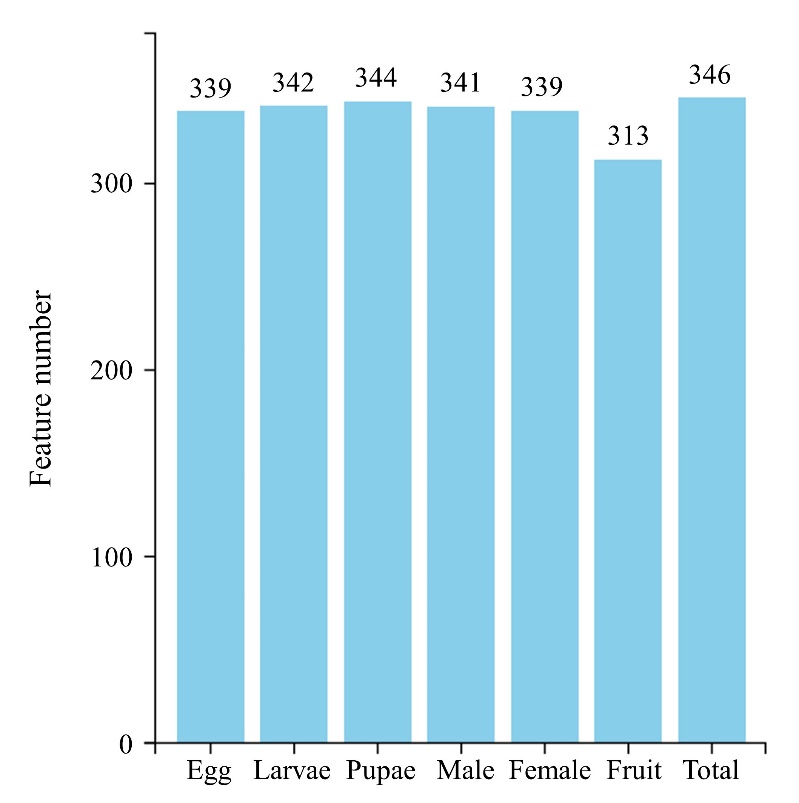


**Fig. S2** Rarefaction curves based on OTU numbers at different developmental stages of *P. okadai* and overripe fruits.


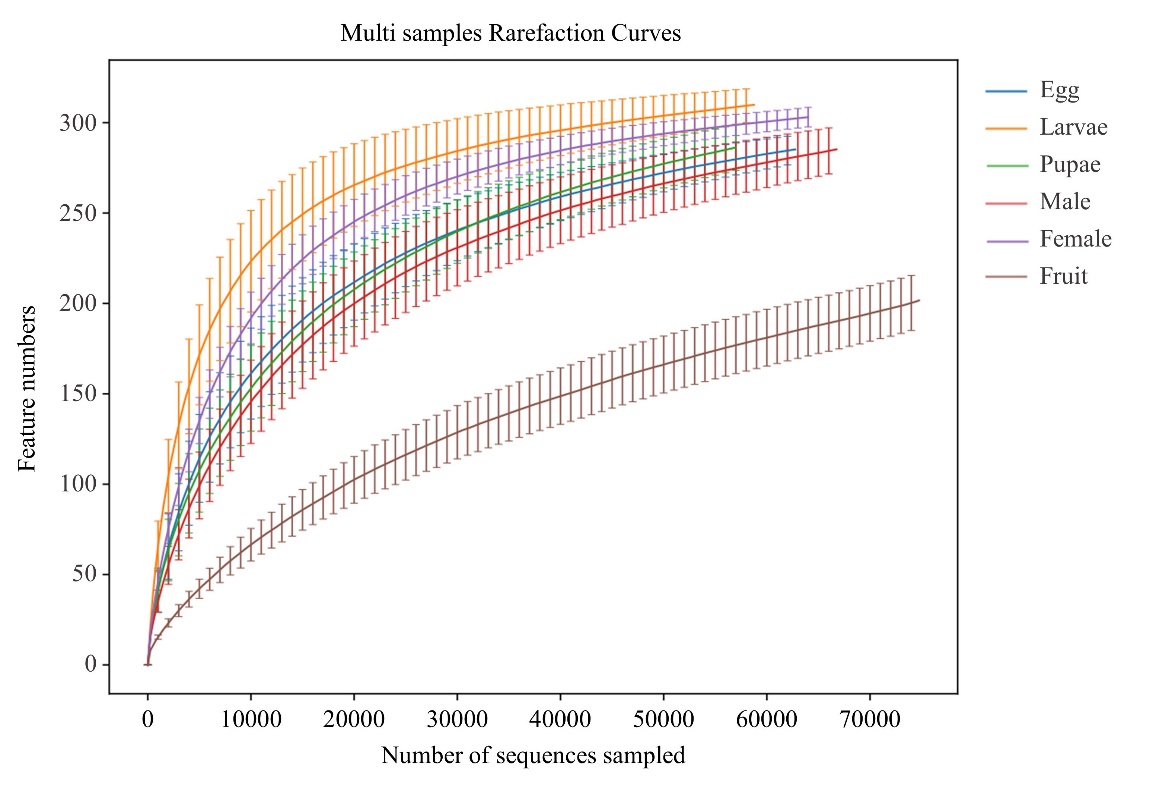


**Fig. S3** Taxonomic composition and relative abundance of symbiotic bacteria at the phylum and genus levels between different developmental stages of *P. okadai* and overripe fruits.


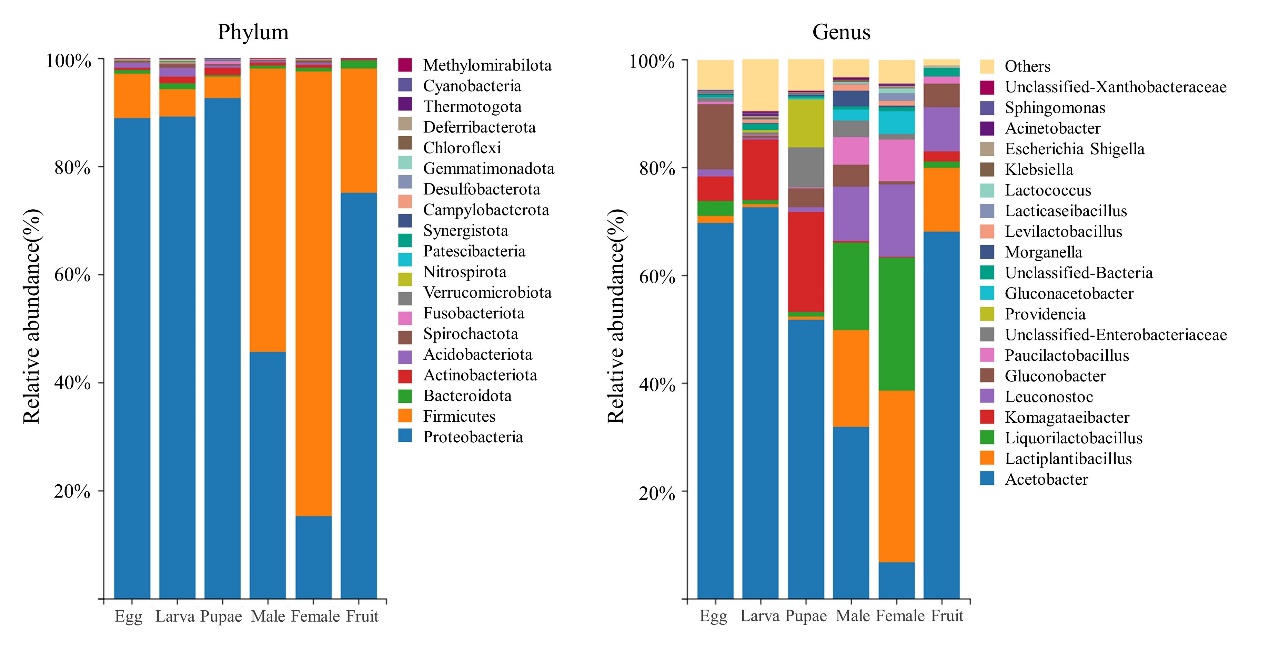


**Fig. S4** Differences in the microbial community abundance of *P. okadai* at different developmental stages and of overripe fruits at the genus level according to analysis of variance (ANOVA, BH-FDR). The figure shows the bacterial genera with the highest percentages of abundance.


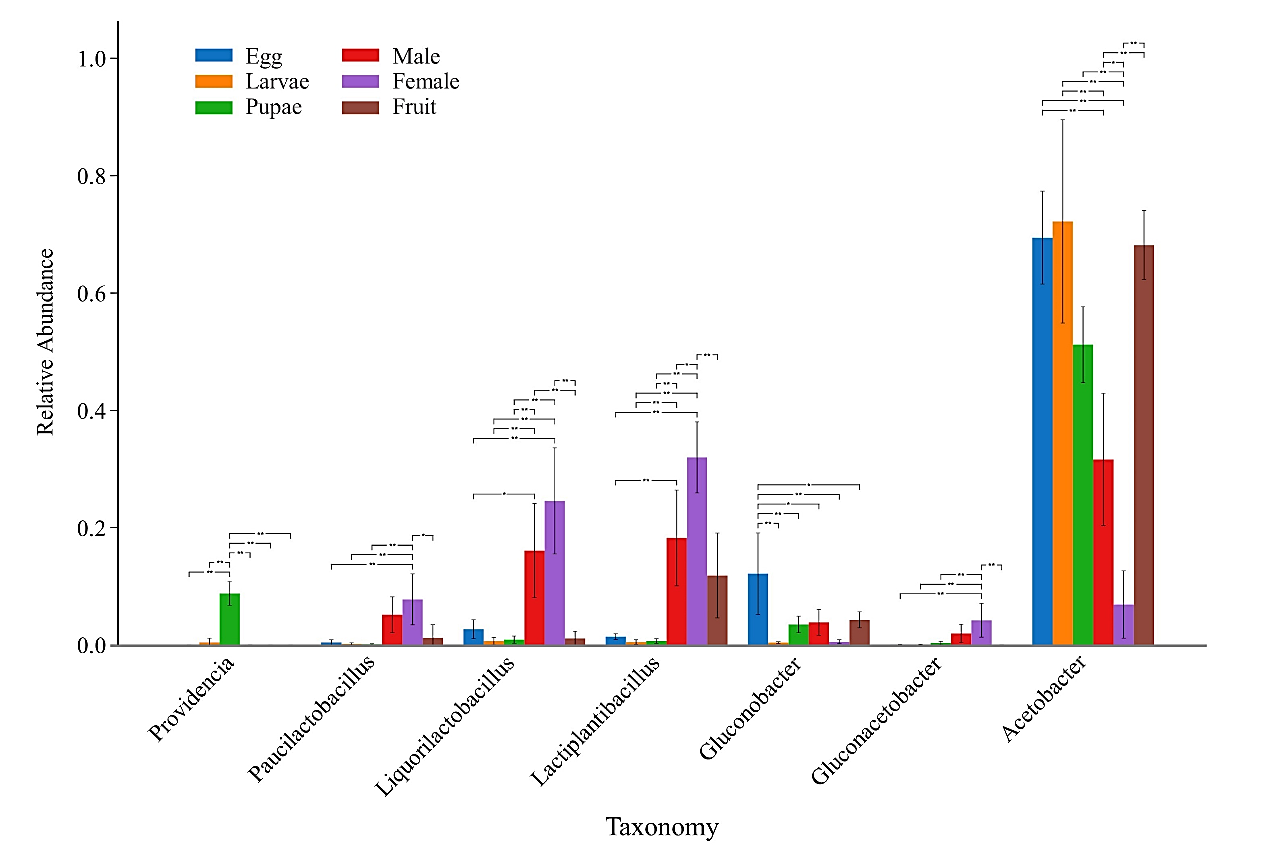


**Table S1** The sequencing data for the overripe fruits and *P. okadai* at different developmental stages in each sample is shown in the following table: raw reads are the number of raw reads obtained from sequencing; clean reads are the number of high-quality reads obtained after raw sequence quality control; effective reads are the number of effective sequences after cleaning reads by splicing (double-end), filter length and chimeras; and AvgLen (bp) is the average sequence length of the sample. (E: egg, L: larvae, P: pupae, M: male midgut, F: female midgut, and S: overripe pear).

| Sample | Raw Reads | Clean Reads | Effective Reads | AvgLen(bp) | Effective(%) |
| --- | --- | --- | --- | --- | --- |
| E1 | 80423 | 80152 | 78549 | 407 | 97.67 |
| E2 | 79879 | 79597 | 77947 | 408 | 97.58 |
| E3 | 80071 | 79815 | 77966 | 407 | 97.37 |
| E4 | 79999 | 79742 | 77391 | 410 | 96.74 |
| L1 | 79504 | 79205 | 77546 | 408 | 97.54 |
| L2 | 80029 | 79763 | 77697 | 409 | 97.09 |
| L3 | 79833 | 79563 | 77873 | 408 | 97.54 |
| L4 | 79844 | 79588 | 77411 | 409 | 96.95 |
| P1 | 80089 | 79803 | 76903 | 410 | 96.02 |
| P2 | 80029 | 79724 | 76219 | 411 | 95.24 |
| P3 | 80227 | 79951 | 77666 | 410 | 96.81 |
| P4 | 79932 | 79628 | 73943 | 413 | 92.51 |
| M1 | 80337 | 80039 | 76853 | 420 | 95.66 |
| M2 | 79989 | 79687 | 76909 | 423 | 96.15 |
| M3 | 80295 | 80049 | 77026 | 417 | 95.93 |
| M4 | 80081 | 79819 | 77623 | 417 | 96.93 |
| F1 | 80001 | 79705 | 77255 | 424 | 96.57 |
| F2 | 79801 | 79508 | 76499 | 423 | 95.86 |
| F3 | 79878 | 79594 | 76717 | 428 | 96.04 |
| F4 | 79999 | 79717 | 77091 | 425 | 96.36 |
| S1 | 79766 | 79542 | 78017 | 410 | 97.81 |
| S2 | 79880 | 79597 | 78244 | 413 | 97.95 |
| S3 | 79999 | 79723 | 78156 | 410 | 97.70 |
| S4 | 79939 | 79661 | 78122 | 410 | 97.73 |

**Table S2** Fifty-four genera were detected in species with a *P. okadai* abundance of more than 99.9%, of which 11 genera are present at different developmental stages and are known as the core microbiota.

| OUT ID | Phylum | Class | Order | Family | Genus |
| --- | --- | --- | --- | --- | --- |
| OUT 1 | Proteobacteria | Alphaproteobacteria | Acetobacterales | Acetobacteraceae | Acetobacter |
| OUT 2 | Firmicutes | Bacilli | Lactobacillales | Lactobacillaceae | Lactiplantibacillus |
| OUT 3 | Proteobacteria | Alphaproteobacteria | Acetobacterales | Acetobacteraceae | Gluconobacter |
| OUT 4 | Proteobacteria | Alphaproteobacteria | Acetobacterales | Acetobacteraceae | Komagataeibacter |
| OUT 5 | Firmicutes | Bacilli | Lactobacillales | Lactobacillaceae | Leuconostoc |
| OUT 6 | Proteobacteria | Gammaproteobacteria | Enterobacterales | Enterobacteriaceae | Uc-Enterobacteriaceae |
| OUT 7 | Firmicutes | Bacilli | Lactobacillales | Lactobacillaceae | Liquorilactobacillus |
| OUT 9 | Firmicutes | Bacilli | Lactobacillales | Lactobacillaceae | Paucilactobacillus |
| OUT 12 | uc-Bacteria | uc-Bacteria | uc-Bacteria | uc-Bacteria | uc-Bacteria |
| OUT 15 | Firmicutes | Bacilli | Lactobacillales | Lactobacillaceae | Liquorilactobacillus |
| OUT 110 | Proteobacteria | Gammaproteobacteria | Enterobacterales | Enterobacteriaceae | Klebsiella |

**Table S3** Relative abundance (%) of taxa associated with overripe fruits and *P. okadai* at different developmental stages at the genus level.

| Taxa | Egg | Larvae | Pupae | Male | Female | Fruits |
| --- | --- | --- | --- | --- | --- | --- |
| Acetobacter | 69.43 | 72.19 | 51.20 | 31.62 | 6.91 | 68.16 |
| Lactiplantibacillus | 1.43 | 0.51 | 0.70 | 18.26 | 31.98 | 11.85 |
| Liquorilactobacillus | 2.72 | 0.68 | 0.92 | 16.09 | 24.57 | 1.14 |
| Komagataeibacter | 4.25 | 11.70 | 18.58 | 0.17 | 0.14 | 1.83 |
| Leuconostoc | 1.38 | 0.23 | 0.89 | 10.25 | 13.10 | 8.29 |
| Gluconobacter | 12.18 | 0.43 | 3.52 | 3.88 | 0.57 | 4.29 |
| Paucilactobacillus | 0.44 | 0.21 | 0.15 | 5.17 | 7.78 | 1.25 |
| Uc_Enterobacteriaceae | 0.74 | 0.35 | 7.55 | 3.23 | 1.11 | 0.14 |
| Providencia | 0.01 | 0.45 | 8.80 | 0.02 | 0.01 | 0.00 |
| Gluconacetobacter | 0.06 | 0.05 | 0.37 | 1.99 | 4.23 | 0.02 |
| Uc_Bacteria | 0.60 | 1.15 | 0.23 | 0.56 | 0.72 | 1.53 |
| Morganella | 0.06 | 0.12 | 0.27 | 2.92 | 0.32 | 0.00 |
| Levilactobacillus | 0.13 | 0.60 | 0.08 | 1.24 | 0.82 | 0.24 |
| Lacticaseibacillus | 0.04 | 0.08 | 0.04 | 0.19 | 1.41 | 0.00 |
| Lactococcus | 0.07 | 0.03 | 0.02 | 0.33 | 1.00 | 0.04 |
| Klebsiella | 0.15 | 0.32 | 0.22 | 0.34 | 0.21 | 0.11 |
| Escherichia_Shigella | 0.12 | 0.29 | 0.19 | 0.08 | 0.15 | 0.03 |
| Acinetobacter | 0.08 | 0.30 | 0.06 | 0.16 | 0.22 | 0.01 |
| Uc_Xanthobacteraceae | 0.12 | 0.33 | 0.08 | 0.11 | 0.16 | 0.02 |
| Sphingomonas | 0.17 | 0.28 | 0.10 | 0.11 | 0.13 | 0.02 |
| Lactobacillus | 0.26 | 0.31 | 0.08 | 0.06 | 0.06 | 0.01 |
| Bacteroides | 0.12 | 0.26 | 0.12 | 0.12 | 0.13 | 0.02 |
| Uc_Muribaculaceae | 0.16 | 0.26 | 0.06 | 0.10 | 0.12 | 0.01 |
| Others | 5.30 | 8.88 | 5.77 | 3.01 | 4.13 | 0.98 |

**Table S4** Alpha diversity of the gut microbiota of *P. okadai* at different developmental stages and overripe fruits. The Chao 1 diversity estimator, the number of OTUs, and the Shannon and Simpson indices were estimated for all the samples (E: eggs, L: larvae, P: pupae, M: male midgut, F: female midgut, S: overripe fruits).

| Sample | ACE | Chao 1 | Shannon | Simpson |
| --- | --- | --- | --- | --- |
| E1 | 327.46 | 321.71 | 1.60 | 0.38 |
| E2 | 310.92 | 315.29 | 2.05 | 0.56 |
| E3 | 322.95 | 325.98 | 1.70 | 0.45 |
| E4 | 304.00 | 304.00 | 2.85 | 0.59 |
| L1 | 324.30 | 330.13 | 1.43 | 0.25 |
| L2 | 329.20 | 333.25 | 2.47 | 0.43 |
| L3 | 323.19 | 324.00 | 1.85 | 0.37 |
| L4 | 330.41 | 355.00 | 2.86 | 0.66 |
| P1 | 321.26 | 325.67 | 2.34 | 0.65 |
| P2 | 323.75 | 321.43 | 2.56 | 0.68 |
| P3 | 331.26 | 328.53 | 2.40 | 0.66 |
| P4 | 342.43 | 343.50 | 3.46 | 0.77 |
| M1 | 312.83 | 311.44 | 3.12 | 0.83 |
| M2 | 318.94 | 318.75 | 3.49 | 0.84 |
| M3 | 331.62 | 343.50 | 3.38 | 0.80 |
| M4 | 314.77 | 329.44 | 2.86 | 0.77 |
| F1 | 306.47 | 311.61 | 3.05 | 0.79 |
| F2 | 320.19 | 320.24 | 3.68 | 0.84 |
| F3 | 315.04 | 321.52 | 2.74 | 0.74 |
| F4 | 314.61 | 315.95 | 3.05 | 0.78 |
| S1 | 361.63 | 287.77 | 1.60 | 0.47 |
| S2 | 295.40 | 297.73 | 2.04 | 0.59 |
| S3 | 258.54 | 229.93 | 1.57 | 0.46 |
| S4 | 273.71 | 263.46 | 1.71 | 0.49 |

**Table S5** BLAST-based alignment of 16S rRNA sequences from the intestinal tract of adult *P. okadai*.

| **Kingdom** | Phylum | Class | Order | Family | Genus | Species | Top-hit strain | Simillary (%) |
| --- | --- | --- | --- | --- | --- | --- | --- | --- |
| Bacteria | Firmicutes | Bacill | Bacillales | Planococcaceae | *Metasolibacillus* | *Lysinibacillus fusiformis* | NBRC 15717 | 99.93 |
| Bacteria | Proteobacteria | Alphaproteobacteria | Rhizobiales | Rhizobiaceae | *Agrobacterium* | *Agrobacterium pusense* | LMG 25623 | 99.78 |
| Bacteria | Firmicutes | Bacilli | Bacillales | Bacillaceae | *Bacillus* | *Bacillus licheniformis* | ATCC 14580 | 99.93 |
| Bacteria | Firmicutes | Bacilli | Bacillales | Bacillaceae | *Fictibacillus* | *Fictibacillus gelatini* | LMG 21880 | 99.93 |
| Bacteria | Firmicutes | Bacilli | Lactobacillales | Lactobacillaceae | *Levilactobacillus* | *Levilactobacillus brevis* | ATCC 14869 | 99.93 |
| Bacteria | Firmicutes | Bacilli | Lactobacillales | Lactobacillaceae | *Leuconostoc* | *Leuconostoc suionicum* | ATCC 8293 | 99.93 |
| Bacteria | Firmicutes | Bacilli | Lactobacillales | Enterococcaceae | *Enterococcus* | *Enterococcus termitis* | LMG 8895 | 99.09 |
| Bacteria | Proteobacteria | Alphaproteobacteria | Rhodospirillales | Acetobacteraceae | *Acetobacter* | *Acetobacter ghanensis* | 430A | 98.90 |
| Bacteria | Proteobacteria | Gammaproteobacteria | Enterobacterales | Morganellaceae | *Morganella* | *Morganella psychrotolerans* | H1r | 99.50 |
| Bacteria | Proteobacteria | Gammaproteobacteria | Pseudomonadales | Pseudomonadaceae | *Entomomonas* | *Entomomonas moraniae* | QZS01 | 99.14 |
| Bacteria | Firmicutes | Bacilli | Lactobacillales | Lactobacillaceae | *Lactiplantibacillus* | *Lactiplantibacillus argentoratensis* | DSM 16365 | 99.86 |
| Bacteria | Proteobacteria | Alphaproteobacteria | Rhodospirillales | Acetobacteraceae | *Acetobacter* | *Acetobacter tropicalis* | NBRC 16470 | 99.71 |
| Bacteria | Proteobacteria | Gammaproteobacteria | Enterobacterales | Enterobacteriaceae | *Citrobacter* | *Citrobacter freundii* | DSM 30039 | 98.44 |
| Bacteria | Firmicutes | Bacilli | Lactobacillales | Lactobacillaceae | *Leuconostoc* | *Leuconostoc citreum* | ATCC 49370 | 99.79 |
